# A comparative study on production of extracellular hydrolytic enzymes of *Candida* species isolated from patients with surgical site infection and from healthy individuals and their co-relation with antifungal drug resistance

**DOI:** 10.1186/s12866-020-02045-6

**Published:** 2020-12-03

**Authors:** Rakhshanda Erum, Farkhunda Samad, Adnan Khan, Shahana Urooj Kazmi

**Affiliations:** Department of Microbiology, University of Karachi, Karachi, 75270 Pakistan

**Keywords:** Surgical site infection, *Candida* species, Antifungal drug resistance, Proteinase, Phospholipase

## Abstract

**Background:**

Surgical site infection (SSI) is a crucial dilemma of surgery. Patients with SSIs not only face difficulty in treatment but also bear extra cost with high mortality rate. Resistant strains of Candida have emerged as an important nosocomial pathogen. Proteinase and phospholipase are exo- enzymes of *Candida* species, have importance with respect to their contribution in diseases. This study focused on prevalence of *Candida* species in surgical wound, their resistance to antifungal drugs, co-relation of these resistance with virulence potential of *Candida* species and comparison of production level of exo-enzymes of *Candida* species isolated from patients with SSIs and healthy individuals to highlights their role in SSIs.

**Results:**

A total of (*n* = 555) swab samples were investigated. (*n* = 450) samples were collected from patients with SSIs and (*n* = 105) were collected from healthy individuals. Samples were subjected for the identification of *Candida* species which were subsequently investigated for antifungal susceptibility, MICs and enzymatic activity of *Candida* species. Out of 128 strains of *Candida* spp. isolated from SSIs, 54(42.18%) were identified as *C. albicans* followed by *C. glabrata* 32(25%), *C. parapsilosis* 17(13.28%), *C. krusei* 13(10.16%) and *C. tropicalis* 12(9.38%). *C. albicans* isolates showed 100% susceptibility to voriconazole and amphotericin B followed by itraconazole 98% and fluconazole 89%. Out of 6 fluconazole resistant *C. albicans* 5(83.33%) were able to produce phospholipase while out of 48 fluconazole-susceptible strains 17(35.42%) were found to be phospholipase producer. Out of 54 *C. albicans* isolated from surgical wound 46(85.18%) and 49(90.74%) were found to be phospholipase and proteinase producer respectively, whereas out of 20 *C. albicans* isolates from healthy subjects 14(70%) produce proteinase and 12(60%) produce phospholipase. There were significant statistical differences found between the level of enzyme production by *C. albicans*, in relation to both sites (*P* = 0.014).

**Conclusion:**

Study revealed that prevalence of *Candida* species is high in SSIs. Phospholipase and proteinase activity were more pronounced in *Candida* Species from surgical wound in contrast to species from healthy individuals suggests these enzymes may have been responsible for the severity of infection in surgical wound patients.

## Background

Surgical site infection (SSI) is one of the postsurgical complications that occur in wound created for surgical purposes. SSIs is defined as the infections caused by pathogenic microorganisms in a wound created by invasive surgical procedure and it can involve tissues, organs, and cavities, involved during surgery [1]. One of the factors for SSIs is the immune status of patient. Patients who have compromised immune system have greater chance to acquire SSIs because of the suppressed immunity, high number of invasive procedures attained and frequent visits to healthcare system. Age and sex of patients, co-morbid conditions such as diabetes mellitus, obesity, lack of nutrition are other factors for SSIs [2]. Among all Patients who encounter surgeries, at least 5% of patients experience this infection [3]. variety of preventive measures such as, careful surgical techniques, appropriate use of prophylactic antibiotics, proper ongoing training for staff and salubrious operating room environment, markedly reduce the chance of surgical site infection [4]. SSIs accounted for one-third of postoperative deaths and 8% of all deaths associated with hospital acquired infections [5]. Patients specially those who belong to low socioeconomic class may have more chance to develop SSIs because of their unhygienic living condition, existence of co-morbidities, inadequate medical assistance, and ignorance in getting medical aid on time [6]. SSIs impact on economy as it increases the length of hospital stay of patient, intensified the treatment expenditure considerably, augmented hospital admission and imperiled the health outcomes [4, 7]. Although many of the guidelines have been developed for prevention of SSIs, these infections still execute substantial burden on surgical patients particularly in low-income countries [8]. *C. albicans* found as normal flora in oral cavities, urogenital and gastrointestinal tracts of healthy people [9] but the ratio of fungi, especially *C. albicans*, is increasing considerably in surgical site infection [10]. Low availability of antifungal agents and improper use of chemotherapeutic agents for longer period as prophylactic drugs alters the microflora of patients which may increases the risk of Candida infection in surgical patients [11]. Due to less antifungal agents available, the treatment for serious *Candida* infections has become difficult. Among few antifungal drugs available, amphotericin B, a polyene fungicidal agent is used as gold standard to treat Candidial infections [12]. Nystatin is also an important drug which is found efficient against several *Candida* species upon in vitro testing [13]. After the introduction of azole antifungal agents, the way to deal with the treatment of serious *Candida* infections has become changed [14]. Azole antifungal compounds have become prime drugs because of their lesser toxicity and utmost efficacy [15]. The first drug included in the azole class is ketoconazole. fluconazole, posaconazole, and voriconazole all are the members of triazole class of antifungal agents which possessed antifungal activity towards *Candida* species in in vitro as well as on clinical basis [16]. For both immunocompetent and immunoompromised patients, fluconazole is a drug of choice, as first line of treatment in infections caused by *Candida* species [17]. Many factors are responsible for emerging resistance against fluconazole such as treatments repetition and exposure of drug for longer period [18]. Apart from genetically determined resistance, *Candida* species have ability to acquire resistance to azole class by three mechanisms [19] that are induction of multi-drug pumps [20, 21], the alteration or up-regulation of the enzyme target lanosterol 14-a-sterol demethylase [22], and the development of bypass pathways [23] which might be the reason of change in susceptibility for each drug within azole class. *Candida* spp. secrete different extracellular enzymes which are proteases, phospholipases and lipases [24]. Phospholipase and proteinase are two putative virulence factors of *Candida* species which are assumed as enhancer of its pathogenicity by accentuating its adhesion, tissue damage, immune system evasion, as well as its dissemination [25]. The extracellular phospholipases act on host cell membrane which results in disruption of host cells or modification of surface attributes that promote adherence and penetration of host cell membranes and ensuing infection [26] while secretory aspartyl proteinases (SAP) are the enzymes of *C. albicans* that have capacity to hydrolyze host proteins such as albumin, immunoglobulin, and skin proteins [24, 27]. Although proteinases are secreted by all *Candida* species but non-albicans *Candida* produce proteinases in very limited level as compared to *C. albicans* [28]. The reason for this less production of proteinase by non-albicans *Candida* as compare to *C. albicans* is not uncovered yet and still under research [29–31]. In order to explore the actual relationship of exo-enzymes of *C. albicans* in surgical site infection, there is need to focus on the level of secretion of these enzymes. This study focused on prevalence of *Candida* species in surgical wound, their resistance to antifungal drugs, co-relation of these resistance with virulence potential of *Candida* species and comparison of production level of two putative extracellular hydrolytic enzymes of *Candida* species isolated from patients with SSIs and from healthy individuals to highlights their role in SSIs.

## Results

### Demographical sketches of patients and analysis of surgical site infection in combination with cause and sampling site

Out of 450 patients 269 (59.77%) were males and 181 (40.22%) were females giving female to male ratio of 1:1.4. The age range of patients was from 9 years to 78 years. The mean age of patients was 31 years (Table 1). Among 450 studied cases 402 were found to be positive for SSIs in which the infection rate was comparatively high 53% in the age group of 20–39 followed by 21% in 40–59 years of age group. Looking into the activities leading to the cause of surgery, disease was the major cause of surgery which accounted for 368 (81.77%) followed by gunshot 58 (12.88%) and accident 24 (5.33%) (Table 2). In association with disease distribution SSIs was most commonly found in patients with intestinal perforation 103 (28%) followed by intestinal obstruction 70 (19%), appendicitis 52 (14%), peritonitis 41 (11%), intestinal hernia 29 (8%), cholecystitis 29 (8%), ulcerative colitis 18 (5%) and others 26 (7%) (Table 3). Patients were also having a number of co-morbidities including diabetes, liver disease and renal disease (Table 4).

**Table 1:** Age and Sex Distribution of Patients with Surgical Site Infection

Age in years	Male (%) (*n* = 269)	Female (%) (*n* = 181)	Patients (n = 450)
0–19	58 (12.88%)	30 (6.66%)	88 (19.55%)
20–39	149 (33.11%)	90 (20%)	239 (53.11%)
40–59	45 (10%)	48 (10.66%)	93 (20.66%)
60–79	17 (3.77%)	13 (3%)	30 (6.66%)

Numbers in parentheses are percentages

**Table 2:** Reason for Surgery

Types of Surgery	Patients (n = 450)	Percentage
Disease	368	81.78
Gunshot	58	12.88
Accident	24	5.34

**Table 3:** Disease Distribution Associated with Surgical Site Infections

Disease	Patients (*n* = 368)	Percentage (%)
Intestinal Perforation	103	28
Intestinal Obstruction	70	19
Appendicitis	52	14
Peritonitis	41	11
Intestinal Hernia	29	8
Cholecystitis	29	8
Ulcerative Colitis	18	5
Others	26	7

**Table 4:** Co-morbid Conditions of Patients with Surgical Site Infection

Diseases	Patients (n = 450)	Percentage (%)
Diabetes	54	12
Mental disorder	11	2.44
Arthritis	27	6
Obesity	18	4
Cardiovascular diseases	14	3.11
Ulcer	7	1.55
No comorbidity	319	70.88

### Pattern of pathogens in pus samples of patients with SSIs

**Fig. 1:**
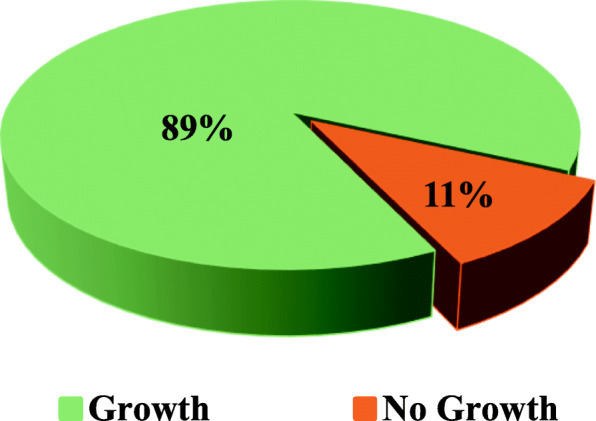
Ratio of Growth of Pathogens Isolated from Patients with SSIs

Out of 450 pus samples obtained from patients with surgical site infections, 402 (89%) samples yielded microbial growth while in 48 (11%) samples no growth observed (Fig. 1), indicative of no surgical site infection. A total of 611 isolates yielded, among which bacterial isolates were 483 (79.05%) while *Candida* species were 128 (20.94%). Culture of the wounds yielded *Escherichia coli* 147 (24%), followed by *Candida* spp. 128 (20.94%), *S. aureus* 110 (18%), *Klebsiella* spp. 98 (16%), *Pseudomonas* spp. 55 (9%), *Proteus* spp. 37 (6%), Coagulase-negative *staphylococci* 21 (3.43%) and others 15 (2.45%) (Table 5).

**Table 5:** Spectrum of Pathogens in Pus Samples of Surgical Wound Patients

Isolates	*n* = 611	Percentage
*Escherichia. Coli*	147	24
*Candida* spp.	128	20.94
*S. aureus*	110	18
*Klebsiella* spp.	98	16
*Pseudomonas* spp.	55	9
*Proteus* spp.	37	6
Coagulase-negative *staphylococci*	21	3.43
Other Pathogens	15	2.45

### Prevalence of *Candida* species in SSIs and healthy individuals

**Fig. 2:**
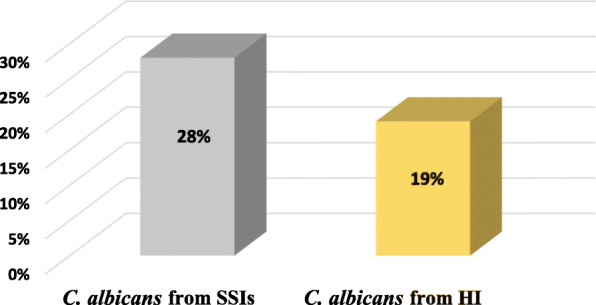
Prevalence of *C. albicans* in Surgical Site Infection (SSIs) and in Healthy Individuals (HI)

Among 450 studied cases of surgical wound infections 128 (28.44%) were found to be positive for *Candida* Species, while in 105 Swab samples from tong dorsum and jugal mucosa of healthy individuals 20 (19.04%) were found to be positive with *C. albicans* by culture (Fig. 2). Out of 128 strains of *Candida* species isolated from patients with SSIs, in comparison to *C. albicans* (*n* = 54)*,* non-*albicans Candida (n =* 74) were predominant. These non-*albicans Candida* (n = 74) were categorized as follows: *C. glabrata* (*n* = 32), *C. parapsilosis* (*n* = 17), *C. krusei* (*n* = 13) and *C. tropicalis* (*n* = 12) (Table 6).

**Table 6:** Spectrum of *Candida* Species in Pus Samples of Surgical Wound Patients

Isolates	*n* = 128	Percentage (%)
*C. albicans*	54	42.18
*C. glabrata*	32	25
*C. parapsilosis*	17	13.28
*C. krusei*	13	10.16
*C. tropicalis*	12	9.38

### Antifungal susceptibility profile of *Candida* spp. isolated from SSIs

Disc diffusion testing of all *Candida* isolates to fluconazole, voriconazole, itraconazole and amphotericin B was performed. *C. albicans* showed 100% susceptibility to voriconazole and amphotericin B followed by itraconazole (98.14%) and fluconazole (88.88%). Moreover, out of 13 *C. kuresi* tested, 12 (92.30%) were susceptible to voriconazole while all 13 strains of *C. krusei* were resistant to fluconazole. Interestingly, *C. parapsilosis* found to be susceptible to all tested drugs (Table 7). The correlation between azole resistance was analyzed statistically. In case of *C. albicans* a significantly higher percentage of isolates had reduced susceptibility to fluconazole than to itraconazole or voriconazole (*P* = 0.010), while in case of *C. glabrata* and *C. tropicalis* no significant values found for these drugs that is (*P* = 0.536) and (*P* = 0.755) respectively.

**Table 7:** Antifungal Susceptibility Profile of *Candida* spp. Isolated from Surgical Wound Patients

*Candida* spp.	Fluconazole		Voriconazole		Itraconazole	
	S	R	S	R	S	R
*C. albicans* (n = 54)	48 (88.88%)	6 (11.11%)	54 (100%)	0 (0%)	53 (98.14)	1 (1.85%)
*C. glabrata* (n = 32)	26 (81.25%)	6 (18.75%)	29 (90.62%)	3 (9.37%)	28 (87.5)	4 (12.5%)
*C. tropicalis* (n = 12)	11 (91.66%)	1 (8.33%)	11 (91.66%)	1 (8.33%)	10 (83.33%)	2 (16.66%)
*C. krusei* (*n* = 13)	0 (0%)	13 (100%)	12 (92.30%)	1 (7.69%)	13 (100%)	0 (0%)
*C. parapsilosis* (n = 17)	17 (100%)	0 (0%)	17 (100%)	0 (0%)	17 (100)	0 (0%)

S Sensitivity, R Resistance, numbers in parentheses are percentages

### Minimum inhibitory concentration (MICs) of antifungal agents for *C. albicans* isolated from SSIs

Additionally, MIC of commonly prescribed antifungal agents was tested in 54 *C. albicans* isolates. Concerning the fluconazole MIC, three *C. albicans* had an MIC of 16 μg/mL. The remaining isolate had MIC 0.125 μg/mL in two isolates, 0.25 μg/mL in five isolates, 0.5 μg/mL in six isolates, 1 μg/mL in three isolates, 2 μg/mL in nine isolates, 4 μg/mL in twelve isolates and 8 μg/mL in fourteen isolates. MIC results of itraconazole showed MIC 0.06 μg/mL in six isolates, 0.125 μg/mL in twenty-one isolates, 0.25 μg/mL in twelve isolates and 0.5 μg/mL in fifteen isolates. When we discuss MIC pattern of voriconazole, we found MIC 0.06 μg/mL in twenty isolates. No interpretative breakpoints have been established for amphotericin B. MICs for amphotericin B were mostly in the range of 0.03—0.25 mg/mL. MIC_50_ and MIC_90_ values for fluconazole were higher than those for the other antifungal agents. MIC_50_ and MIC_90_ of fluconazole were 2 and 8 μg/mL respectively while for itraconazole and amphotericin B MIC_50_ and MIC_90_ were 0.125 μg/mL and 0.5 μg/mL and 0.06 and 0.25 μg/mL respectively. MIC_50_ of voriconazole was 0.06 μg/mL while MIC_90_ of this drug for *C. albicans* was 0.5 μg/mL (Table 8).

**Table 8:** MICs of Antifungal Agents against *C. albicans* Isolated from Surgical Wound Patients

Antifungal drugs	MIC Range (μg/ml)	MICs μg/ml		Resistant strains%
		MIC 50 MIC90		
Fluconazole	0.125–16	2	8	6
Voriconazole	0.03–0.5	0.06	0.5	0
Itraconazole	0.06–0.5	0.125	0.5	0
Amphotericin B	0.03–0.25	0.06	0.25	0

### Manifestation of enzymatic activity of *Candida* species isolated from patients with SSIs and healthy individuals

**Fig. 3:**
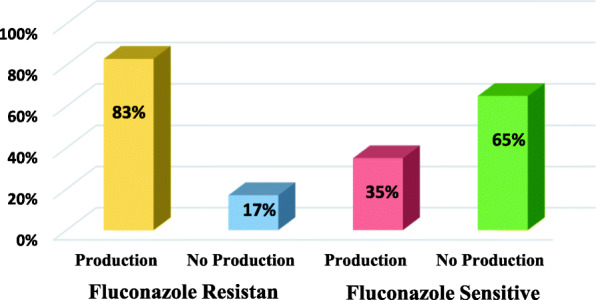
Phospholipase Production in Fluconazole Resistant and Fluconazole Sensitive Strains of *C. albicans*

The phospholipase and proteinase activity were more pronounced in *Candida albicans* in contrast to non-*albicans Candida* (Table 9)*.* Among all phospholipase producing non-albicans *Candida*, *Candida krusei* (46.15%) were found to be high phospholipase producer followed by *C. glabrata* (31.25%), *C. tropicalis* (25.0%) and *C. parapsilosis* (5.88%) (Table 10). Out of 54 *C. albicans* isolated from surgical wound 46 (85.18%) were found to be phospholipase producers while the proteinase production rate was found in 49 (90.74%) of isolates. Out of 20 *C. albicans* isolates from healthy subjects14 (70%) produce proteinase and 12 (60%) produce phospholipase (Table 11). The enzymatic activity was measured by dividing colony diameter to the diameter of the precipitation zone (Pz) around the colony formed on the plate. A Pz (in mm) of 1.0 was evaluated as negative (−), 0.99–0.9 as weak (+), 0.89–0.8 as mild (++), 0.79–0.7 as relatively strong (+++) and 0.69-below (++++) as very strong positive. *C. albicans* demonstrated high proteinase activity, with Pz values varying from 0.17 to 0.90 for the surgical wound isolates and from 0.44 to 0.96 for the isolates of healthy subjects. *C. albicans* isolates showed high or low phospholipase activity, with Pz values varying from 0.39 to 0.96 for surgical wound isolates and from 0.57 to 0.93 for the isolates of healthy individuals. The high activity of phospholipases for *C. albicans* was observed in 54.34% of the surgical wound isolates and 25.0% in isolates of healthy individual. There were significant statistical differences (*P* = 0.014) between the patterns of enzyme production by *Candida albicans* isolated from patients with SSIs and healthy individuals (Table 12). We also evaluated the phospholipase production between fluconazole-resistant and fluconazole-susceptible strains of *C. albicans* in order to explore the relationship between resistance to antifungal drugs and virulence of *C. albicans*. Out of 6 fluconazole resistant *C. albicans* 5 (83.33%) were able to produce phospholipase while out of 48 fluconazole-susceptible strains 17 (35.42%) were found to be phospholipase producer (Fig. 3).

**Table 9:** Prevalence of Phospholipase and Protienase Producing *Candida* species in Surgical Wound Patients

Extracellular enzymes	*Candida albicans* (n = 54)	Non-albicans *Candida* (n = 74)
Phospholipase	46 (85.18%)	20 (27.02%)
Proteinase	49 (90.74%)	52 (70.27%)

**Table 10:** Production of Phospholipase in non-albicans *Candida* species

Non-albicans *Candida* (n = 74)	Phospholipase producing strains (*n* = 20)
*C. glabrata* (n = 32)	**10 (31.25%)**
*C. parapsilosis* (n = 17)	**1 (5.88%)**
*C. krusei* (n = 13)	**6 (46.15%)**
*C. tropicalis* (n = 12)	**3 (25.0%)**

**Table 11:** Phospholipase and Proteinase Activity of *C. albicans* Isolates from Patients with Surgical Wound and Healthy Individuals

*Candida albicans*	Producers	Non producers	Pz Ranged
Surgical wound (n = 54)			
Proteinase	49 (90.74%)	5 (9.25%)	0.17–0.81
Phospholipase	46 (85.18%)	8 (14.81%)	0.39–0.82
Healthy subjects (n = 20)			
Proteinase	14 (70.0%)	6 (30.0%)	0.44–0.62
Phospholipase	12 (60.0%)	8 (40.0%)	0.57–0.93

**Table 12:** Enzymatic Activity (mm) Exhibited by *C. albicans* Isolated from Patients with Surgical Wound and Healthy Individuals

Pz Value		Phospholipase		Proteinase	
		Surgical wound isolates (n = 54)	Healthy Individual Isolates (n = 20)	Surgical wound isolates (n = 54)	Healthy Individual Isolates (n = 20)
< 0.69	++++	25 (54.34)	3 (25.0)	34 (69.38)	2 (14.28)
0.70–0.79	+++	11 (23.91)	5 (41.66)	8 (16.32)	5 (37.71)
0.80–0.89	++	6 (13.04)	3 (25.0)	5 (10.20)	4 (28.57)
0.90–0.99	+	4 (8.69)	1 (8.33)	2 (4.08)	3 (21.42)
1.00	–	8 (14.81)	8 (40.0)	5 (9.25)	7 (30.0)

Pz Value = Enzymatic activity zone, Numbers in parentheses are percentages

## Discussion

Surgical site infection (SSI) constitutes a major complication after surgery [32] which still stands as the most frequent form of undesirable hospital events [33]. Despite the development in infection control practices, the incidence of SSIs is still increasing, especially in low and middle-income countries [34]. There are limited data available focusing the incidence of SSIs in Pakistan. One of the prospective studies conducted in Pakistan by Sangrasi et al. [35] revealed that surgical site infections causes considerable morbidity and economic burden. Another local study conducted on surveillance of SSIs, documented that the surgical wound infections rates are much higher than the National Nosocomial Infection Surveillance (NNIS) standards [36]*.*

In this study incidence of SSIs was higher in males. This predominance is might be due to more exposure of males in surrounding for their work, and for other activities when compared with females thus they have more risk of accidental injuries [37, 38]. In the present study the infection rate was comparatively high (53.11%) in the age group of 20–39 while a lower percentage (6.66%) was seen in 60–79 year age group. Similar findings regarding the age distribution of patients with SSIs was found in other studies [38]. In this study, disease was the major cause of surgery and SSIs were most commonly found in patients with intestinal perforation (28%) followed by intestinal obstruction (19%), appendicitis (14%), peritonitis (11%), intestinal hernia (8%), cholecystitis (8%), ulcerative colitis (5%) and others (7%). Similar findings were observed by Mawalla et al. in Tanzania where they also found high rate (27%) of SSIs in patients with peritonitis, intestinal obstruction and intestinal perforation, (15%) of patients with appendicitis and (14.3%) patients with cholecystitis also had SSIs [37]. Another study also highlights the fact that SSIs was observed in 22% cases of incisional hernia and 7% cases of bowel obstruction [39]. In our study, among patients with SSIs 131 patients having pre-morbid illnesses of which 54 (12%) were suffering from diabetes. Comparable findings have been accrued by other investigators [37, 40] and they reported that patients having pre-morbid health problems, such as diabetes are at high risk of appearing SSIs, because their defense system have been compromised as compared to healthy individuals.

450 pus samples isolated from patients with SSIs were analyzed. The growth positivity was observed in 89.33% of samples. This high rate of growth positivity could be because of specimens were collected from patients who have sign and symptoms indicative of surgical site infections. Our results are in accordance with study conducted by Giacometti et al. [41] who worked on epidemiology and microbiology of SSIs and they also found growth in 90.82% of samples.

Our findings showed *E. coli* (24%) were the most isolated pathogen of surgical site infection. Several studies conducted in different parts of the world also reported *E. coli* as the main culprit of surgical site infection [42–44]. The possible explanation for *E. coli* most frequently isolated in this study is may be due to the fact that most of the specimens were taken from patients who underwent abdominal surgeries [45]. This can be due to incidental spilling of bowel flora during surgery.

*Candida* species are found as normal flora of human skin, genital and oral mucosa as well as gastrointestinal area [46]. Healthy people have 3–47% of *Candida* species as oral normal flora [47]. Candida has emerged as an important nosocomial pathogen from the last few years [8, 48]. As there is a lack of studies focusing the impact of colonization of *Candida* species as a risk factor for SSIs, therefore the correlation between the *Candida* colonization as a risk factor for SSIs is still questionable. We are unaware of any previous study from Pakistan addressing the contribution of the *Candida* species to the risk of surgical site infection in patients undergoing surgeries. This study focused on prevalence of *Candida* species in surgical site infection, their resistance to antifungal drugs, co-relation of these resistance with virulence potential of *Candida* species and comparison of production level of two putative exo-enzymes, phospholipase and proteinase of *Candida* species isolated from patients with SSIs and from healthy individuals in order to highlights their role in SSIs. Our data demonstrated the clinical significance of *Candida* colonization. According to our data, prevalence of *Candida* species in SSIs was (28.4%) which is in accordance with study conducted in Poland [49] where they also found *Candida* species (29%) in surgical wound patients. In addition, the result of this study contrast to study conducted in Nigeria [50] where they found *Candida* infection in 9% patients with SSIs. Presence of *Candida* spp. in surgical wound is not unusual happening because the prolonged used of chemotherapy alters the microbial flora of surgical patients which may increases the chance of *Candida* infection. In the present study, *C. albicans* followed by *C. glabrata* were the most commonly isolated *Candida* species. Li and YZ [51] documented the same pattern of *Candida* spp. in patients of surgical intensive care unit. This substantial percentage of *C. albicans* demonstrating the role of fungi in surgical wound and it is an alarming bell for doctors as well as for individuals associated with health care providing services.

The increasing resistance to antifungal agents has aroused the requirement of an antifungal sensitivity testing to treat patients with fungal infections. Antifungal susceptibility of *Candida* species was performed against fluconazole, voriconazole, itraconazole and amphotericin B. *C. albicans,* showed 100% susceptibility to voriconazole and amphotericin B followed by itraconazole (98.14%) and fluconazole (88.88%). These findings are in accordance with study conducted by Citak et al., and Badiee and Alborzi [52, 53] who reported resistance to fluconazole 87.5 and 89.5% respectively. Furthermore, several studies conducted in Europe, South America and the USA demonstrated that, before the year of 2005 the rate of resistance to fluconazole and itraconazole was very less in nosocomial isolates [54], but it was gradually increased during the latter five years of the decade not only against azoles but also against echinocandins [55]. In addition, this study revealed, in non-*albican Candida* strain, *C. glabrata* (19%) were resistant to fluconazole followed by *C. tropicalis* (8%). These findings are consistent with study [56] but in inconsistent to local study conducted by Farooqi et al. [57] where they reported 0% resistance to these drugs. The diverse capacity of *C. albicans* strains to adapt to antifungal exposure [58] and mutation are might be the reasons for antifungal resistance especially in clinical isolates. Another possible reason for this increasing ratio of resistance to antifungal drugs in *Candida* spp. is the extensive and long-term use as well as the application of short courses of the antifungal agents for treatment. Moreover, all tested strains of *C. krusei* were resistant to fluconazole. *C. krusei* is usually intrinsically resistant to fluconazole, [59]. Orozco et al. who investigated the three general mechanisms of fluconazole resistance in *C. krusei* reported that the predominant mechanism of fluconazole resistance in *C. krusei* is a 14α-demethylase with reduced susceptibility to the inhibitory effects of fluconazole [60].

Additionally, MICs of commonly prescribed antifungal agents was determined for 54 *C. albicans* isolates. In case of fluconazole, (5.55%) strains showed MICs at 16 μg/mL which is 2 times greater than breakpoint levels. These finding showed higher MIC value in comparison to MIC observed by Pfaller et al. [61] in USA where they found the fluconazole MIC for *C. albicans* at 0.5–2.0 μg/mL. Furthermore, (27.77%) strains of *C. albicans* showed MIC at 0.5 μg/mL towards itraconazole comparable to studies conducted in USA [62] where they observed MIC at 0.5 μg/mL in 5% clinical isolates of *C. albicans*. This high percentage indicated that resistance to itraconazole is increasing in *C. albicans.* MIC_50_ and MIC_90_ of fluconazole were 2 μg and 8 μg/mL respectively which are similar with observations of other investigators [63]. In case of voriconazole, MIC_50_ and MIC_90_ were 0.06 μg/mL and 0.5 μg/mL. Mandras et al. [56] also reported similar findings for MIC_50_ but they found MIC_90_ at 0.12 μg/mL.

*Candida* species are commensal of host epithelial tissues that usually reside as normal flora in oral cavities, urogenital and gastrointestinal tracts of healthy people. One of the aims of this study was to highlights the role of extracellular hydrolytic enzymes of *Candida albicans* in SSIs. To achieve this goal, we focused on different parameters which effects on the secretion level of these enzymes. One of the parameters was pH. To compare the secretion levels of extracellular hydrolytic enzymes of *C. albicans* isolated from patients with SSIs and healthy individuals we have taken samples from the oral route because the pH of mouth is neutral or near to neutral [64] while the pH of vagina is acidic and on acidic pH these enzymes may trigger to secrete [65].

The findings of the current study revealed that the phospholipase and proteinase activity were more pronounced in *C. albicans* as compared to non-*albicans Candida.* The proteinase and phospholipase production were observed 49/54 (90.74%) and 46/54(85.18%) in *C. albicans* isolates, followed by non-*albicans Candida* species 52/74 (70.27) and 20/74 (27.02) respectively. These results are in accordance with study conducted by Kumar et al. [31] who worked on *Candida species* recovered from HIV seropositive and cancer patients, reported that enzymatic activity was high in *C. albicans* with 100% phospholipase and 94.1% proteinase activity as compared to *Candida non-albicans* species with 29.6% phospholipase and 70.3% proteinase activity. The findings of this study are also consistent with study conducted by Jasim et al. [66] who studied the virulence factors of *Candida* species isolated from clinical specimens and they observed proteinase-producing ability in 31 (79.5%) *C. albicans* isolates followed by non-*C. albicans albicans* 7(63.63%) and Sachin et al. [30] who reported the high proteinase production in *C. albicans* (82.1%) followed by *Candida non albicans* (80%); which were isolated from different clinical specimens. The reason for this less production of proteinase by non-albicans Candida as compare to *C. albicans* is not uncovered yet and still under research [29–31]. The variance in the virulence attributes of *Candida albicans* may depend on the type, site and stage of infection and the immune status of patients [67]. For the comparison of virulence property of *C. albicans* isolated from surgical wound and healthy control, oral swabs were collected from healthy individuals. The oral samples from SSI patients were not taken for this study because surgery patients have compromised immune system and on antibiotic treatment, so it might be possible that their normal oral flora also be effected and might affect the secretion of relative level of hydrolytic enzymes of *Candida albicans*.

Another reason for not taken oral samples from SSI Patients, as this study was done in Pakistan which is geographically located in South Asia where over one-third of tobacco consumed regionally is smokeless and Traditional forms like betel quid, tobacco with lime and tobacco tooth powder are commonly used not only among men but also among children, teenagers and women of reproductive age [68] which influence on normal oral flora and change the microbial ecosystem. The alteration in the microflora in any way either by the immune suppression or by the use of smokeless tobacco can lead to the growth and proliferation of pathogenic microorganisms such as *Candida albicans* which is a component of normal oral flora but they switch to pathogenic form [69] and starts to release their enzymes more pronouncedly. The patients (*n* = 239) in this study belonged to 20–39 years of age group and majority were habitual of smokeless tobacco (Paan and Gutka). As our aim was to highlights the role of extracellular hydrolytic enzymes of *Candida* Species in surgical wound infections so it was necessary to take samples from those who have healthy and natural oral conditions so that we were be able to present the exact comparison of the secretion of these enzymes in healthy and infection conditions. Among 105 healthy individuals, 19.04% were found to be positive with *C. albicans* while non-albicans *Candida* species were not detected in any of volunteer*.*

These findings of current study are consistent with studies conducted in India [70] and Brazil [71] where they also found the prevalence rate of oral *C. albicans* 15, 17, and 26% respectively. However, a study conducted in France by Sitterle et al. who screened 56 undergraduate students to evaluate the prevalence of oral *Candida* carriage in healthy individuals reported that 10 of the 56 students (17.9%) were carriers of *Candida* spp., 8 harboured only *C. albicans*, and 2 harboured both *C. albicans* and *C. glabrata* [72]. In this study we observed that proteinase activity of *C. albicans* isolated from surgical wound and healthy individuals were (90.74%) and (70.27%) while the phospholipase activity was (85%) and (60%) respectively which suggest that these enzymes may have been responsible for the severity of infection in surgical wound patients. Pinto et al. [73] who worked on patients with denture-related stomatitis and control individuals reported that the phospholipase activity of *Candida* spp. was higher in infection as compared to commensal. Furthermore, a study conducted by Borst and Fluit who worked on differences in secretion level of two putative virulence factors of *Candida albicans* isolated from different sites of infection reported that, *Candida* Species that isolated from respiratory infections secreted phospholipase and proteinase in a considerable amount as compare to species isolated from wounds, blood, and the urinary tract [74]. Such disparity might be because of factors such as origin of isolates, the vast phenotypic variability of the isolate, or possibly a variance in the technique used.

With a purpose to investigate the co-relation between resistance to antifungal agents and virulence of *C. albicans*, we observed the level of phospholipase production in fluconazole- resistant and fluconazole- susceptible strains of *C. albicans*. We found majority of (83.33%) fluconazole resistant *C. albicans* were able to produce phospholipase while only (35.42%) fluconazole-susceptible strains found to be phospholipase producer. These findings are in accordance to the observations of Ying and Chunyang [75] who also reported that high phospholipase production correlate with fuloconazole resistance. Forgacs et al. [76] used two *C. albicans* strains (a fluconazole-sensitive clinical isolate and a fluconazole-resistant laboratory mutant) to examine the changes in virulence traits accompanying the development of resistance to fluconazole and they stated that the fluconazole-resistant strains proved to be superior in the virulence traits examined.

## Conclusion

Despite the fact that surgical site infection constitutes a major complication after surgery, there is still lack of data that describes its epidemiology. A considerable deliberation is required to a more definite comprehension of the SSIs. The higher prevalence of *Candida* species among surgical wound patients may be related to increased infection in this group of patients which may results in increased morbidity and mortality by delayed wound healing. The development of resistance to fluconazole has become a matter of concern as it contributes in an increased virulence in *Candida* species. Phospholipase and proteinase activity were more pronounced in *Candida* Species from surgical wound in contrast to species isolated from healthy individuals, highlights the role of these enzyme in SSIs as an enhancer of the pathogenic potentials of *Candida* species.

## Methods

### Study population

450 Patients who underwent surgeries and developed any signs and symptoms indicative of surgical site infections were selected for the study. Swab samples of tong dorsum and jugal mucosa were also collected from 105 healthy volunteers as control. A proper approval from the ethical committee, University of Karachi with approval number IBC-005-13-16 have been obtained for the study and consent was taken from all the patients prior the sample collection.

### Collection of samples

A. Samples from Patients with Surgical Site Infections

Samples were taken form 450 patients having surgical site infection with sign and symptoms including redness, warmth, and pain. Other symptoms include extreme tenderness at surgical site, purulent discharge, increased body temperature and swelling of wounded area. Two pus swabs were taken from every patient with the help of sterile swab and transported to research laboratory within 1 h for further processing.

B. Samples from Healthy Individuals

Swab samples of tong dorsum and jugal mucosa were collected from 105 healthy adults who were apparent good conditions of oral hygiene, not immunocompromised, and were not currently hospitalized. Test people were asked for to forgo brushing their teeth for the 24-h period going before examination. After collection, samples were transported to laboratory to examine the existence of *C. albicans* by standard procedures.

### Isolation and identification

For the growth of yeast species, samples were cultured on Sabrouad’s dextrose agar (SDA) (Oxoid, Basingstoke,UK) supplemented with 50 mg/L of chloramphenicol and incubated at 30 °C for 48 h at static condition. Yeast isolates were then subjected to mycological identification by germ tube test, chlamydospore formation on corn meal and rice agar, biggy agar, carbohydrate assimilation test and opacity-test in Tween 80-CaCl_2_ agar. After presumptive identification, isolated colonies with indicative phenotype of *C. albicans* were investigated for growth at 45 °C on modified Sabouraud’s glucose agar (SGA) to facilitate the differentiation of *C. albicans* and *C. dubliniensis*. Isolation and Identification of bacterial pathogens were also performed using standard microbiological methods.

### Antifungal susceptibility of *Candida* species by disc diffusion method

Antifungal susceptibility of *Candida* spp. was measured by disc diffusion method following the document M44-A, proposed by CLSI. Firstly, test suspension was prepared. The turbidity of suspension was adjusted to 0.5 McFarland standards. Subsequently suspension was inoculated on Mueller Hinton agar (MHA) supplemented with 2% glucose and 0.5 μg/mL of methylene blue.

Plates of MHA were leaved for 5–15 min to dry. Antifungal discs, flucnazole (25 μg), voriconazole (1 μg) were obtained from Oxide and amphotericin B (100 μg) and itraconazole (10 μg) were prepared by filter paper. After placing the discs on agar, plates were incubated at 35 °C for 24 and 48 h. Inhibitory zone diameters were measured at the transitional point where growth abruptly decreased, as determined by a marked reduction in colony sizes and interpreted by standard interpretive criteria. *C. albicans* (ATCC90029), *C. prapsilosis* (ATCC 22019), and *C. krusei* (ATCC6258) were used as quality control strains.

### MICs of antifungal agents for *C. albicans* by microdilution method

Sensitivity profile of *C. albicans* to different antifungal drugs including fluconazole, amphotericin B, voriconazole and itraconazole were assessed by broth microdilution test according to CLSI. Test suspensions were prepared by suspending 4 to 5 colonies of *C. albicans* in 0.9% saline and turbidity was adjusted to 0.5 McFarland standards with approximately 1–5 × 10^6^ CFU/mL. Di-methyl sulfoxide (DMSO) or water was used to prepared solutions of drugs. Two-fold serial dilutions of antifungal agents were prepared with RPMI 1640 containing L-glutamine without bicarbonate, buffered to pH 7.0 with 0.165 M morpholinepropane sulfonic acid (MOPS; Sigma). Final concentrations of fluconazole ranged from 0.125 to 64 μg/mL and voriconazole, amphotericin B, and Itraconazole from 0.03 to 16 μg/mL. The susceptibility assays were performed in sterile 96-well microplates. To achieve a final test volume of 200 μL, 100 μL of antifungal drug (from final concentration) and 100 μL suspension of test isolates was added to each well. Two wells were run as positive and negative control wells. In positive control well only the yeast suspension was added while the negative control well lacks the yeast suspension and only had the drug suspension. The volumes of both control wells were adjusted to a final test volume (200 μL) by sterile saline. The plates were incubated at 37 °C for 48 h. Plates which have the amphotericin B drug were wrapped using aluminum foil to protect them from light. After 24 h, the MIC value was recorded as the least concentration of antifungal drug that inhibited at least 80% of the growth of organism as compared to positive control well. The results were noted not only as the least and the highest MIC value but additionally as the values of MIC_50_ and MIC_90_. The MIC_50_ and MIC_90_ values expressed that specific concentration of drug which has potential to suppress the growth of isolates to 50 and 90% respectively. In this test the strain of *C. parapsilosis* ATCC 22019 was used as control strain.

### Preparation of yeast suspension for enzymatic activity

To prepare yeast suspension a well isolated colony of *Candida* species was picked from primary isolation plates and suspended in a sterile saline. The turbidity of suspension was adjusted to 0.5 McFarland (1 × 10^8^ CFU/mL).

### Determination of enzymatic activity by agar plate method

#### Phospholipase activity

The egg yolk agar plate method described by Price et al. [77] was used to detect phospholipase activity of *Candida* species isolated from surgical wounds and healthy subjects. The Test medium used to detect phospholipase enzyme was consisted of agar 20 g, peptone 10 g, sodium chloride 57.3 g, calcium chloride 0.55 g, glucose 30 g and sterile egg yolk enrichment (50%) 100 mL/1000 mL of distilled water. 10 μL suspension of test isolate was inoculated on agar plate. After inoculation, the plates were incubated at 37 °C for 48 h *C. albicans* ATCC 10231 used as positive controls.

Formation of an opaque zone (precipitation of a calcium complex) around the *Candida* colony was identified as phospholipase activity. The zone of precipitation was calculated using the method depicted by Price et al. [77]. According to that method, ratio of the diameter of colony to the total diameter of colony plus zone of precipitation (Pz) was considered as the zone of precipitation of the enzyme tested.

$$ \mathrm{Pz}=\frac{\mathrm{Diameter}\ \mathrm{of}\ \mathrm{colony}}{\mathrm{Total}\ \mathrm{diameter}\ \mathrm{of}\ \mathrm{colony}\ \mathrm{plus}\ \mathrm{zone}\ \mathrm{of}\ \mathrm{precipitation}}. $$

Depending on this method, Pz = 1.00 suggests that the test strain is negative for phospholipase, while Pz = 0.63 implies that the test strain is releasing considerable quantity of phospholipase. Estimations of Pz in between 0.64 and 0.99 showed that the test strain is releasing small amounts of phospholipase.

#### Proteinase activity

To verify the enzymatic activity of proteinase [78], bovine serum albumin (BSA) agar was used which was composed as follows: BSA 2 g, yeast nitrogen base (YNB) (Difco Laboratories) 145 g, glucose 20 g and agar 20 g/1000 mL of distilled water. 10 μL of test suspension containing 1 × 10^8^ CFU/mL was inoculated on test medium. The plates were incubated at 37 °C for 72 h for proteinases. A clear halo around each colony were measured as proteinase activity and used in the determination of the precipitation zone (Pz) values. *C. albicans* ATCC 10231 used as positive controls. The proteinase activity was determined in a similar manner as delineated for phospholipase.

### Statistical analysis

All data were analyzed in the statistical packages for social science (SPSS-19). Frequency and percentage were computed for qualitative observation using chi-square test and fisher exact test and the chi-square test was also used to analyze the correlation between different azole drugs. *P* < 0.05 was considered as significant.

## Data Availability

The datasets used and/or analyzed during the current study are available from the corresponding author on reasonable request.

## Abbreviations

SSIs: Surgical site infections
CA: *Candida albicans*
NAC: Non-albicans Candida
SAP: Secretory aspartyl proteinases
SDA: Sabrouad’s dextrose agar
Pz: Precipitation zone
YNB: Yeast nitrogen base
NNIS: National Nosocomial Infection Surveillance

## References

[1] Bagnall NM, Vig S, Trivedi P (2009). Surgical-site infection. Surgery (Oxford).

[2] Emori TG, Gaynes RP (1993). An overview of nosocomial infections, including the role of the microbiology laboratory. Clin Microbiol Rev.

[3] Smyth ET, McIlvenny G, Enstone JE (2008). Four country healthcare associated infection prevalence survey 2006: overview of the results. J Hosp Infect.

[4] Reichman DE, Greenberg JA (2009). Reducing surgical site infections: a review. Rev Obstet Gynecol.

[5] Coello R, Charlett A, Wilson J, Ward V, Pearson A, Borriello P (2005). Adverse impact of surgical site infections in English hospitals. J Hosp Infect..

[6] Triantafyllopoulos G, Stundner O, Memtsoudis S, Poultsides LA (2015). Patient, surgery, and hospital related risk factors for surgical site infections following Total hip Arthroplasty. ScientificWorldJournal..

[7] Broex ECJ, Van Asselt ADI, Bruggeman CA, Van Tiel FH (2009). Surgical site infections: how high are the costs?. J Hosp Infect..

[8] Thomas TA (2017). WHO guidelines to prevent surgical site infections (for low – and middle – income countries). Curr Med Issues.

[9] Bai FY (2014). Association of genotypes with infection types and antifungal susceptibilities in *Candida albicans* as revealed by recent molecular typing strategies. Mycology..

[10] Jarvis WR (1995). Epidemiology of nosocomial fungal infections, with emphasis on Candida species. Clin Infect Dis.

[11] Azevedo MM, Teixeira-Santos R, Silva AP (2015). The effect of antibacterial and non-antibacterial compounds alone or associated with antifugals upon fungi. Front Microbiol.

[12] Ostrosky-Zeichner L, Rex JH, Pappas PG, Hamill RJ, Larsen RA, Horowitz HW (2003). Antifungal susceptibility survey of 2,000 bloodstream *Candida* isolates in the United States. Antimicrob Agents Chemother.

[13] Arikan S, Ostrosky-Zeichner L, Lozano-Chiu M, Paetznick V, Gordon D, Wallace T (2002). In vitro activity of nystatin compared with those of liposomal nystatin, amphotericin B, and fluconazole against clinical *Candida* isolates. J Clin Microbiol.

[14] Saag MS, Dismukes WE (1988). Azole antifungal agents: emphasis on new triazoles. Antimicrob Agents Chemother.

[15] Mohamed SA, Al-Ahmadey ZZ (2013). Biofilm formation and antifungal susceptibility of *Candida* isolates from various clinical specimens. Br Microbiol Res J.

[16] Chen A, Sobel JD. Emerging azole antifungals. Expert Opin. Emerg. Drugs. 2005;10(1):21–33. 10.1517/14728214.10.1.21 15757401

[17] Meis J, Petrou M, Bille J, Ellis D, Gibbs D (2000). A global evaluation of the susceptibility of *Candida* species to fluconazole by disk diffusion. Diagn Microbiol Infect Dis.

[18] Enwuru CA, Ogunledun A, Idika N, Enwuru NV, Ogbonna E, Aniedobe M, Adeiga A (2008). Fluconazole resistant opportunistic oro-pharyngeal candida and non-candida yeast-like isolates from HIV infected patients attending ARV clinics in Lagos, Nigeria. Afr Health Sci.

[19] Kanafani ZR, Perfect JR (2008). Resistance to antifungal agents: mechanisms and clinical impact. Clin Infect Dis.

[20] Albertson GD, Niimi M, Cannon RD, Jenkinson HF (1996). Multiple efflux mechanisms are involved in Candida albicans fluconazole resistance. Antimicrob Agents Chemother.

[21] Sanglard D, Ischer F, Monod M, Bille J (1997). Cloning of Candida albicans genes conferring resistance to azole antifungal agents: characterization of CDR2, a new multidrug ABC transporter gene. Microbiology.

[22] Loffler J, Kelly SL, Hebart H, Schumacher U, Lass-Florl C, Einsele H (1997). Molecular analysis of cyp51 from fluconazole-resistant Candida albicans strains. FEMS Microbiol Lett.

[23] Kelly SL, Lamb DC, Kelly DE (1997). Resistance to fluconazole and cross-resistance to amphotericin B in Candida albicans from AIDS patients caused by defective sterol delta5,6-desaturation. FEBS Lett.

[24] Chaffin WL (2008). *Candida albicans* Cell Wall proteins. Microbiol Mol Biol Rev.

[25] Nidhi P, Munesh KG, Ragini T (2018). Extracellular hydrolytic enzyme activities of the different *Candida* spp. isolated from the blood of the intensive care unit-admitted patients. J Lab Physicians.

[26] Barrett-Bee KE, Hayes Y, Wilson RG, Ryley JF (1985). A comparison of phospholipase activity, cellular adherence and pathogenicity of yeasts. Microbiology..

[27] Cassone A, Bernardis FD, Mondello F, Ceddia T, Agatensi L (1987). Evidence for a correlation between proteinase secretion and vulvovaginal candidosis. J Infect Dis.

[28] Rüchel R, De Bernardis F, Ray TL, Sullivan PA, Cole GT (1992). *Candida* acid proteinases. J Med Vet Mycol.

[29] Jeffery-Smith A, Taori SK, Schelenz S, Jeffery K, Johnson EM, Borman A, Manuel R, Brown CS (2018). Candida auris: a review of the literature. Clin Microbiol Rev.

[30] Sachin CD, Ruchi K, Santosh S (2013). *In vitro* evaluation of proteinase, phospholipase and haemolysin activities of *Candida* species isolated from clinical specimens. Int J Med Biomed Res.

[31] Kumar CPG, Kumar SSJ, Menon T (2006). Phospholipase and proteinase activities of clinical isolates of *Candida* from immunocompromised patients. Mycopathologia..

[32] Webster J, Osborne S (2012). Preoperative bathing or showering with skin antiseptics to prevent surgical site infection. Cochrane Database Syst Rev.

[33] Lewis SS, Moehring RW, Chen LF, Sexton DJ, Anderson DJ (2013). Assessing the relative burden of hospital-acquired infections in a network of community hospitals. Infect Control Hosp Epidemiol.

[34] Stewart B, Khanduri P, McCord C, Ohene-Yeboah M, Uranues S, Vega Rivera F (2014). Global disease burden of conditions requiring emergency surgery. Br J Surg.

[35] Sangrasi AK, Leghari AA, Memon A, Talpur AK, Qureshi GA, Memon JM (2008). Surgical site infection rate and associated risk factors in elective general surgery at a public sector medical university in Pakistan. Int Wound J.

[36] Pishori T, Siddiqui AR, Ahmed M (2003). Surgical wound infection surveillance in general surgery procedures at a teaching hospital in Pakistan. Am J Infect Control.

[37] Mawalla B, Mshana SE, Chalya PL, Imirzalioglu C, Mahalu W (2011). Predictors of surgical site infections among patients undergoing major surgery at Bugando medical Centre in Northwestern Tanzania. BMC Surg.

[38] Bajracharya A, Agrawal A, Yam B, Agrawal C, Lewis O (2010). Spectrum of surgical trauma and associated head injuries at a university hospital in eastern Nepal. J Neurosci Rural Pract.

[39] Murray BW, Cipher DJ, Pham T, Anthony T (2011). The impact of surgical site infection on the development of incisional hernia and small bowel obstruction in colorectal surgery. Am J Surg.

[40] Delamaire M, Maugendre D, Moreno M, Le Goff MC, Allannic H, Genetet B (1997). Impaired leucocyte functions in diabetic patients. Diabet Med.

[41] Giacometti A, Cirioni O, Schimizzi AM, Del Prete MS, Barchiesi F, D'errico MM (2000). Epidemiology and microbiology of surgical wound infections. J Clin Microbiol.

[42] Verma AK, Kapoor AK, Bhargava A (2012). Antimicrobial susceptibility pattern of bacterial isolates from surgical wound infections in tertiary Care Hospital in Allahabad, India. Internet J Med Update-EJOURNAL.

[43] Schnüriger B, Inaba K, Eberle BM, Wu T, Talving P, Bukur M, Belzberg H, Demetriades D (2010). Microbiological profile and antimicrobial susceptibility in surgical site infections following hollow viscus injury. J Gastrointest Surg.

[44] Sani RA, Garba SA, Oyewole OA (2012). Antibiotic resistance profile of gram negative bacteria isolated from surgical wounds in Minna, Bida, Kontagora and Suleja areas of Niger state. Am J Med Med Sci.

[45] Montravers P, Gauzit R, Muller C, Marmuse JP, Fichelle A, Desmonts JM (1996). Emergence of antibiotic-resistant bacteria in cases of peritonitis after intraabdominal surgery affects the efficacy of empirical antimicrobial therapy. Clin Infect Dis.

[46] Sardi JCO, Scorzoni L, Bernardi T, Fusco-Almeida AM, Giannini MM (2013). *Candida* species: current epidemiology, pathogenicity, biofilm formation, natural antifungal products and new therapeutic options. J Med Microbiol.

[47] Samaranayake LP, MacFarlane TW, Lamey PJ, Ferguson MM (1986). A comparison of oral rinse and imprint sampling techniques for the detection of yeast, coliform and *Staphylococcus aureus* carriage in the oral cavity. J Oral Pathol.

[48] Costa SF, Marinho I, Araujo EAP, Manrique AEI, Medeiros EAS, Levin AS (2000). Nososcomial fungaemia: a 2-year prospective study. J Hosp Infect.

[49] Wroblewska MM, Swoboda-Kopec E, Rokosz A, Krawczyk E, Marchel H, Luczak M (2002). Epidemiology of clinical isolates of Candida albicans and their susceptibility to triazoles. Int J Antimicrob Agents.

[50] Isibor JO, Oseni A, Eyaufe A, Osagie R, Turay A (2008). Incidence of aerobic bacteria and Candida albicans in post-operative wound infections. Afr J Microbiol Res.

[51] Li S, An YZ (2010). Retrospective analysis of invasive fungal infection in surgical intensive care unit. Zhonghua Yi Xue Za Zhi.

[52] Citak S, Ozcelik B, Cesur S, Abbasoglu U (2005). In vitro susceptibility of Candida species isolated from blood culture to some antifungal agents. Jpn J Infect Dis.

[53] Badiee P, Alborzi A. Susceptibility of clinical Candida species isolates to antifungal agents by E-test, Southern Iran: A five year study. Iran J Microbiol. 2011;3(4):183–88. PMC3330181 22530086

[54] Pfaller MA, Diekema DJ (2007). Epidemiology of invasive candidiasis: a persistent public health problem. Clin Microbiol Rev.

[55] Pfaller MA, Moet GJ, Messer SA, Jones RN, Castanheira M (2011). *Candida* bloodstream infections: comparison of species distributions and antifungal resistance patterns in community-onset and nosocomial isolates in the SENTRY antimicrobial surveillance program, 2008-2009. Antimicrob Agents Chemother.

[56] Mandras N, Tullio V, Allizond V, Scalas D, Banche G, Roana J (2009). In vitro activities of fluconazole and voriconazole against clinical isolates of *Candida* spp. determined by disk diffusion testing in Turin, Italy. Antimicrob Agents Chemother.

[57] Farooqi JQ, Jabeen K, Saeed N, Iqbal N, Malik B, Lockhart SR (2013). Invasive candidiasis in Pakistan: clinical characteristics, species distribution and antifungal susceptibility. J Med Microbiol.

[58] Jensen RH, Astvad KMT, Silva LV (2015). Stepwise emergence of azole, echinocandin and amphotericin B multidrug resistance in vivo in *Candida albicans* orchestrated by multiple genetic alterations. J Antimicrob Chemother.

[59] Krcmery V, Barnes AJ (2002). Non-albicans *Candida* spp. causing fungaemia: pathogenicity and antifungal resistance. J Hosp Infect..

[60] Orozco AS, Higginbotham LM, Hitchcock CA (1998). Mechanism of fluconazole resistance in Candida krusei. Antimicrob Agents Chemother.

[61] Pfaller MA, Messer SA, Hollis RJ, Jones RN, Doern GV, Brandt ME (1999). Trends in species distribution and susceptibility to fluconazole among blood stream isolates of *Candida* species in the United States. Diagn Microbiol Infect Dis.

[62] Pfaller MA, Messer SA, Hollis RJ, Jones RN (2001). In vitro activities of posaconazole (Sch 56592) compared with those of itraconazole and fluconazole against 3,685 clinical isolates of *Candida* spp. and *Cryptococcus neoformans*. Antimicrob Agents Chemother.

[63] Kalkanci A, Berk E, Aykan B, Caglar K, Hizel K, Arman D (2007). Epidemiology and antifungal susceptibility of *Candida* species isolated from hospitalized patients. J Med Mycol.

[64] Baliga S, Muglikar S, Kale R (2013). Salivary pH: a diagnostic biomarker. J Indian Soc Periodontol.

[65] Carvalho-Pereira J, Vaz C, Carneiro C, Pais C, Sampaio P (2015). Genetic variability of Candida albicans Sap8 propeptide in isolates from different types of infection. Biomed Res Int.

[66] Jasim ST, Flayyih MT, Hassan AA (2016). Isolation and identification of Candida spp. from different clinical specimens and study the virulence factors. World J Pharm Pharmaceut Sci.

[67] Naglik JR, Challacombe SJ, Hube B (2003). *Candida albicans* secreted aspartyl proteinases in virulence and pathogenesis. Microbiol Mol Biol Rev.

[68] Gupta PC, Ray CS (2003). Smokeless tobacco and health in India and South Asia. Respirology..

[69] Patil S, Rao RS, Sanketh DS, Amrutha N (2013). Microbial flora in oral diseases. J Contemp Dent Pract.

[70] Anila K, Hallikeri K, Shubhada C, Naikmasur VG, Kulkarni RD (2011). Comparative study of *Candida* in oral submucous fibrosis and healthy individuals. Revista Odonto Ciência.

[71] de Azevedo Izidoro ACS, Semprebom AM, Baboni FB, Rosa RT, Machado MAN, Samaranayake LP (2012). Low virulent oral *Candida albicans* strains isolated from smokers. Arch Oral Biol.

[72] Sitterlé E, Maufrais C, Sertour N (2019). Within-host genomic diversity of *Candida albicans* in healthy carriers. Sci Rep.

[73] Pinto E, Ribeiro IC, Ferreira NJ, Fortes CE, Fonseca PA, Figueiral MH (2008). Correlation between enzyme production, germ tube formation and susceptibility to fluconazole in *Candida* species isolated from patients with denture-related stomatitis and control individuals. J Oral Pathol Med.

[74] Annemarie B, Ad CF (2003). High levels of hydrolytic enzymes secreted by Candida Albicans isolates involved in respiratory infections. J Med Microbiol.

[75] Ying S, Chunyang L (2012). Correlation between phospholipase of *Candida albicans* and resistance to fluconazole. Mycoses.

[76] Fekete-forgács K, Gyüre L, Lenkey B (2000). Changes of virulence factors accompanying the phenomenon of induced fluconazole resistance in *Candida albicans*. Mycoses..

[77] Price MF, Wilkinson ID, Gentry LO (1982). Plate method for detection of phospholipase activity in *Candida albicans*. Sabouraudia..

[78] Sardi JC, Duque C, Höfling JF, Gonçalves RB (2012). Genetic and phenotypic evaluation of *Candida albicans* strains isolated from subgingival biofilm of diabetic patients with chronic periodontitis. Med Mycol.

